# Ibrutinib Inhibits Angiogenesis and Tumorigenesis in a BTK-Independent Manner

**DOI:** 10.3390/pharmaceutics14091876

**Published:** 2022-09-05

**Authors:** Jia Liu, Zhuojun Liu, Jing Zhang, Xiaofang Chen, Junge Chen, Linlin Sui, Jian Yu

**Affiliations:** 1 School of Engineering Medicine, Beihang University, Beijing 100083, China; 2 School of Biological Science and Medical Engineering, Beihang University, Beijing 100083, China; 3 Beijing Advanced Innovation Center for Biomedical Engineering, Beihang University, Beijing 100083, China; 4 Core Lab Glycobiol & Glycoengn, College of Basic Medical Sciences, Dalian Medical University, Dalian 116000, China

**Keywords:** Ibrutinib, Acalabrutinib, Zanubrutinib, vascular endothelial dysfunction, angiogenesis inhibitor

## Abstract

BTK inhibitor (BTKi) Ibrutinib carries an increased bleeding risk compared to more selective BTKis Acalabrutinib and Zanubrutinib, however, its impact on vascular endothelium remains unknown. In this study, we found that Ibrutinib induced stronger cytotoxic effect on endothelial cells than Zanubrutinib, however, Acalabrutinib cytotoxicity was extremely weak. RNA-seq, followed by KEGG analysis and quantitative RT-PCR validation, was conducted to identify the differential apoptotic target genes of BTKis, leading to their distinct cytotoxic effects on endothelial cells, which showed that Ibrutinib and Zanubrutinib dramatically modulated the expression of critical apoptotic genes, *GADD45B*, *FOS*, and *BCL2A1*, among which *FOS* and *GADD45B* were upregulated more significantly by Ibrutinib than Zanubrutinib, however, Acalabrutinib downregulated *BCL2A1* moderately and was not able to modulate the expression of *FOS* and *GADD45B*. Next, we performed in vitro angiogenesis assays and found that Ibrutinib was more able to induce endothelial dysfunction than Zanubrutinib via stimulating more BMP4 expression, however, Acalabrutinib had no such effect. Especially, the capacity of Ibrutinib to induce endothelial dysfunction can be antagonized by targeting BMP4. Accordingly, Ibrutinib, as an angiogenesis inhibitor, inhibited ovarian and breast cancer progression in vivo. Collectively, our findings addressed a novel molecular basis underlying Ibrutinib-induced endothelial cell dysfunction and suggested the potential application of Ibrutinib to treat angiogenesis-dependent cancers.

## 1. Introduction

Bruton tyrosine kinase (BTK), a key component of B-cell receptor (BCR), plays a prominent role in the survival of many B-cell malignancies, and has thereby become an attractive target for the treatment of B-cell malignancies, such as chronic lymphocytic leukemia (CLL), mantle-cell lymphoma (MCL), Waldenström’s Macroglobulinemia (WM) and diffuse large B cell lymphoma (DLBCL) [1,2,3]. Ibrutinib is the first-class BTKi approved by FDA in 2013, binding to Cys-481 residue in the ATP binding domain of BTK irreversibly, and thereby impairing tumor-cell survival via inhibiting BCR signaling [4]. Acalabrutinib and Zanubrutinib are the second-generation covalent BTKis approved by FDA in 2017 and 2019 respectively, binding to Cys-481 residue of BTK irreversibly with more selectivity [5,6]. All of these three BTKis are well tolerance with rapid and endurable responses, however, they have some adverse events clinically such as bleeding, which could be partially due to the bystander effects on targets other than BTK, especially Ibrutinib [5,6,7]. Early data indicate that the bleeding events occurring in patients taking Ibrutinib are up to 50% [8], while it is rare in patients taking Acalabrutinib [9] or Zanubrutinib [6,10].

Previous findings indicated that the higher bleeding rates of Ibrutinib might be caused by platelet dysfunction [11,12]. BTK and TEC are expressed in human platelets and involved in platelet activation through mediating signaling from collagen receptor glycoprotein VI (GPVI) [13], whose deficiency is associated with bleeding phenotypes in mice or humans [8,14], and TEC could regulate platelet activation in the absence of BTK [15,16]. Ibrutinib, Acalabrutinib and Zanubrutinib have comparable efficiencies to inhibit BTK, however, the IC50 value of Ibrutinib against TEC is lower than Acalabrutinib and Zanubrutinib [9,17,18], which may account for the higher rates of Ibrutinib to induce bleeding events clinically. Furthermore, Ibrutinib can also inhibit SFK (SRC family kinase), a platelet kinase phosphorylating several signaling molecules in the GPVI signaling pathway, whose inhibition would cause hemostatic dysfunction and increase bleeding risk, while Acalabrutinib has no such effect on SFK [12].

Even though blockage of platelet signaling may play an important role in bleeding events associated with Ibrutinib treatment, bleeding events might not happen while the functional morphology of blood vessels is maintained [19]. Considering that the destroyed junction between adjacent vascular endothelial cells may cause bleeding events [20,21], we hypothesized that vascular endothelial damage may exist in bleeding events associated with Ibrutinib treatment. In this study, we for the first time illustrated the high capacity of Ibrutinib to induce apoptosis and dysfunction of endothelial cells versus Acalabrutinib and Zanubrutinib and performed transcriptome-wide RNA sequencing analyses to reveal the molecular basis underlying such effects. Our findings also proposed the potential strategy to relieve endothelial cell dysfunction associated with Ibrutinib treatment by targeting BMP4, and application of Ibrutinib for the treatment of angiogenesis-dependent cancers, as a new angiogenesis inhibitor.

## 2. Materials and Methods

### 2.1. Cell Culture and Reagents

Umbilical cord samples were obtained from full-term deliveries (*n* = 3) and primary human umbilical vein endothelial cells (HUVECs) were isolated and then cultured in EGMTM-2 Endothelial Cell Growth Medium-2 BulletKitTM (#CC-3162, Lonza, Germany). Full-term deliveries provided umbilical cord samples after they signed consent forms, which were approved by the Institutional Review Board at Beihang University and in compliance with the Declaration of Helsinki. We obtained JeKo-1, Mino and Jurkat cell lines from ATCC and such cells were grown in RPMI1640 (Hyclone, Waltham, MA, USA) respectively, containing 10% fetal bovine serum (FBS; GIBCO, Carlsbad, CA, USA), 100 μg/mL streptomycin, 100 U/mL penicillin and 2 mM L-glutamine. The SK-OV-3 (human ovarian cancer) and BCAP-37 (human breast cancer) cells were purchased from ATCC and Cell Bank of the Chinese Scientific Academy respectively, which were cultured in RPMI-1640 complete medium (10% FBS). Cell-cultures were maintained at 37 °C in a humidified atmosphere containing 5% CO_2_. Ibrutinib (HY-10997), Acalabrutinib (HY-17600) and Zanubrutinib (HY-101474) were obtained from MedChem Express (Shanghai, China). Human recombinant BMP4 (rhBMP4; #314-BP) and anti-BMP4 mAb (#MAB757) were purchased from R&D Systems (Shanghai, China).

### 2.2. In Vitro Angiogenesis Assay

Tube formation assay, a model for assessment of angiogenesis in vitro, was conducted as previously described [22]. Briefly, a 24-well plate was coated with Matrigel (#356231) from BD Biosciences by adding 300 μL of ice-cold Matrigel solution per well, and maintained at 37 °C for 30 min in a humidified atmosphere of 5% CO_2_. HUVECs were then seeded on the Matrigel-coated wells (1 × 10^5^ cells/well). Cells were treated with Ibrutinib, Acalabrutinib, Zanubrutinib, rhBMP4 (200 ng/mL) and/or anti-BMP4 mAb (2 μg/mL), and then maintained at 37 °C in a humidified atmosphere of 5% CO_2_. Cells were stained with Calcein AM (#1915662) from Invitrogen after 8 h incubation, and the tube formation was observed under fluorescence microscopy (4 × objective). The parameters of detected networks were analyzed using ImageJ software (Angiogenesis Analyzer).

### 2.3. Cell-Viability Assay

AlamarBlue assay was designed to examine the viability of HUVECs according to manufacturer’s protocol (Bio-Rad, Serotec, Oxford, UK). Cells were seeded in 96-well culture plates (BD Biosciences, San Jose, CA, USA) at 5 × 10^3^ cells/100 μL/well (*n* = 5). AlamarBlue solution (10 μL/well) was added to each well and the plates were continuously incubated for additional 3 h. Fluorescence values at a 560 nm excitation and 590 nm emission wavelengths were measured using the Infinite E Plex Microplate reader (Tecan, Männedorf, Switzerland), and were used to calculate the cell viability based on manufacturer’s manual.

### 2.4. Quantitative RT-PCR (qRT-PCR)

Total RNA was extracted with TRIzol (#15596018; Thermo Fisher Scientific, Beijing, China). RNA samples meeting the following requirements were used in subsequent experiments: RNA integrity number (RIN) > 7.0 and a 28S:18S ratio > 1.8. RNA samples were treated with RNase-Free DNase (Invitrogen) to avoid genomic DNA contamination following the manufacturer’s instructions. Superscript III First-strand Synthesis System (#18080-051; Invitrogen, Beijing, China) was used to synthesize cDNA from 1 μg of total RNA according to the manufacturer’s protocols. qRT-PCR was performed using PowerUp SYBR Green Mix (#00710493; Applied Biosystems, Beijing, China). The primer sequences used for qRT-PCR were shown in Supplementary Table S1.

### 2.5. Immunoblot Analysis

RIPA buffer (#89900; Thermo Fisher Scientific) and Protease/Phosphatase Inhibitor Cocktail (#5872; Cell Signaling Technology, Danvers, MA, USA) were used to prepare cell lysates at 4 °C. Protein concentrations were quantified using BCA Protein Assay Kit (#23227; Thermo Fisher Scientific) based on the manufacturer’s instructions. Equal amounts of protein were applied to a 10% polyacrylamide gel and transferred to a polyvinylidene difluoride membrane (#IPVH00010) from Immobilon-P after electrophoresis. BTK (#8547; 1:1000), BMP4 (#4680; 1:1000), cleaved PARP (#5625; 1:1000), cleaved caspase-3 (#9661; 1:1000) and GAPDH (#5174, 1:3000) were obtained from Cell Signaling Technology. SuperSignal West Femto Maximum Sensitivity Substrate (#34095; Thermo Fisher Scientific) was used to detect chemiluminescence signals. Images were captured by Mini Chemiluminescent Imaging and Analysis System (Sage Creation Science, Beijing, China). The integrated optical density (IOD) of bands was analyzed by Gel-Pro Analyzer 4.0 software (Media Cybernetics, Rockville, MD, USA).

### 2.6. RNA-Seq Analysis

Trizol reagent (Invitrogen) was used to harvest the treated and untreated cells. The total RNA extraction operation was the same as the qRT-PCR section. CapitalBio Technology (Beijing, China) generated and sequenced the sequence libraries. Independent libraries were constructed for triplicate samples of all assays, and then were sequenced on an Illumina HiSeq sequencer (Illumina). |log2FC| ≥ 1 (FC: fold change of expressions) in the transcript abundance and q ≤ 0.05 were the parameters for classifying significantly differentially expressed genes (DEGs). KEGG pathway enrichment analysis was performed for the DEGs using the Goseq R package and KOBAS 3.0 software (Available online: http://kobas.cbi.pku.edu.cn, accessed on 18 December 2021), and *p*-values < 0.05 were considered to be significant. RNA-seq data are accessible at NCBI (BioProject accession number: PRJNA608627).

### 2.7. Apoptosis Analysis

FITC Annexin V Apoptosis Detection Kit I (#556547, BD Biosciences, San Jose, CA, USA) was used to detect the cell apoptotic rate, according to the manufacturer’s instructions. HUVECs were harvested with Trypsin–EDTA (0.25%), centrifuged at 1100 rpm for 5 min and washed with cold PBS. The cells were resuspended in 100 μL Annexin V binding buffer diluted with 1:10 and stained with Annexin V-FITC (5 μL) and PI (3 μL) for 15 min at room temperature in the dark. Then, 400 μL of Annexin V binding buffer diluted with 1:10 was added and the cells were analyzed by flow cytometry. The percentages of Annexin V/PI positive cells were evaluated, based on quadrants determined from single-stained and unstained control samples.

### 2.8. Xenograft Model Analysis

All animal experiments were conducted in accordance with the guidelines provided by the Animal Care and Use Committee of Dalian Medical University. A total of 20 BALB/c nude mice (6 weeks old) were obtained from Dalian Laboratory Animal Co., Ltd. (Dalian, China) and used in this study. The mice were bred under specific pathogen-free conditions. Ovarian cancer cells, SK-OV-3, and breast cancer cells, BCAP-37, were subcutaneously inoculated into the back of mice (10 mice/cell line) next to the right front limb (1 × 10^7^ cells/mouse). Mice were randomly assigned to 2 treatment groups: vehicle and ibrutinib (5 mice/group). Ibrutinib was injected by the intraperitoneal route at a dose of 10 mg/kg every 2 days beginning on day 8 after tumor implantation. The mice were observed, and the tumor volume was measured using a standard caliper every 2 days. On day 21 (SK-OV-3) and 15 (BCAP-37), xenografted tumor tissues were collected, and their weight and volume were measured prior to further histological evaluation. Tumor volume (V) was calculated as follows: V = 0.5 × L × W^2^, where L and W are defined as the tumor length (L) and width (W), respectively. The tumor tissues were dissected for immunohistochemistry analysis.

### 2.9. Immunohistochemistry (IHC)

Tumor tissue samples were fixed with formalin (10%) and embedded with paraffin. For IHC staining, the samples were treated with 5% BSA at room temperature for 1 h before they were cut into 5 μm sections. Blocked sections were then treated with CD31 (#77699, Cell Signaling Technology, USA) or BMP4 (#12492-1-AP, Proteintech, Beijing, China) primary antibody at 4 °C on a shaker overnight. The sections were washed with PBS twice and incubated at room temperature for 2 h with secondary antibodies. After washing with PBS again, sections were incubated with HRP (Dako REAL Ebision kit; DAKO, Glostrup, Denmark) based on the manufacturer’s protocol.

### 2.10. Statistical Analysis

Analyses for significance were performed with GraphPad Prism 8.0 (GraphPad Software Inc., San Diego, CA, USA). Data were presented as mean ± SEM. Differences between 2 groups were determined by unpaired 2-tailed Student’s t-test. Differences among multiple groups were determined by 1-way or 2-way ANOVA with Tukey’s multiple comparisons test. *p*-values < 0.05 were considered significant.

## 3. Result

### 3.1. BTKis Differentially Induce Apoptosis in Endothelial Cells

**Figure 1:**
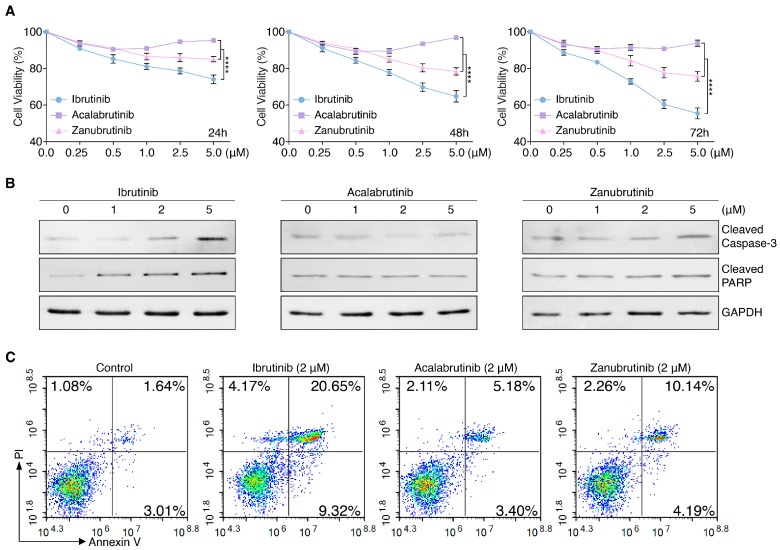
BTKis induce caspase-dependent apoptosis in HUVECs. (**A**) BTKis reduce cell-viability of HUVECs. Endothelial cells were treated with Ibrutinib, Acalabrutinib or Zanubrutinib in a dose-dependent manner for 24, 48 and 72 h (*n* = 6). The cell-viability was determined by AlamarBlue assay. Data are shown as mean ± SEM; **** *p* < 0.0001, as calculated using two-way ANOVA with Tukey’s multiple comparisons test. (**B**) The levels of cleaved caspase-3 and cleaved PARP were increased in endothelial cells treated with Ibrutinib or Zanubrutinib, and remained constant after treatment with Acalabrutinib in a dose-dependent manner for 24 h, which were examined by immunoblot analysis. (**C**) Flow cytometry analysis of apoptosis induced by Ibrutinib, Acalabrutinib or Zanubrutinib at 48 h in HUVECs stained with Annexin V-FITC and PI, and analyzed by NovoExpress software. The percentages in the top right and bottom right of each pseudocolor plot indicate the proportion of Annexin V/PI-positive apoptotic cells.

Considering the adverse drug reactions in patients under treatment of BTKis, especially Ibrutinib, and the association of vascular endothelial cell damage with bleeding, we examined the angiogenesis impact of Ibrutinib, Acalabrutinib and Zanubrutinib on the endothelium. HUVECs were treated with Ibrutinib, Acalabrutinib or Zanubrutinib individually compared to control for 24, 48, and 72 h in a dose-dependent manner, followed by AlamarBlue cell-viability assay. The results showed that Ibrutinib induced significantly stronger anti-angiogenesis effect on endothelial cells than Zanubrutinib, however, Acalabrutinib had very weak suppression to endothelial cells (Figure 1A). Immunoblot analysis results showed a higher level of cleaved caspase-3 and cleaved PARP in HUVECs treated with Ibrutinib or Zanubrutinib compared to untreated cells, which were not changed significantly in HUVECs treated with Acalabrutinib. (Figure 1B and Supplementary Figure S1A). The flow cytometry results revealed that Ibrutinib increased the apoptosis ratio significantly, compared with Acalabrutinib and Zanubrutinib at 48 h (Figure 1C, Supplementary Figure S1B,C).

### 3.2. RNA-Seq Identifies BTKi-Regulated Apoptotic Genes

**Figure 2:**
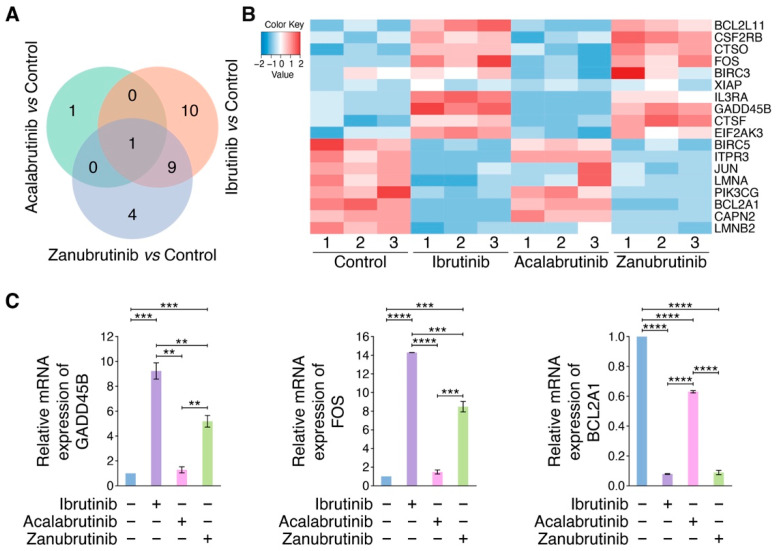
RNA-seq identifies apoptotic genes regulated by BTKis. (**A**) Venn diagram illustrating the number of genes involved in apoptosis, which were commonly and differentially regulated by the treatment of Ibrutinib, Acalabrutinib or Zanubrutinib compared to control treatment in HUVECs. (**B**) Heatmap of DEGs implicated in apoptosis regulated by Ibrutinib, Acalabrutinib and Zanubrutinib versus control in HUVECs. Red represents upregulation and blue indicates downregulation. (**C**) Analysis of mRNA expression of *GADD45B*, *FOS* and *BCL2A1* in endothelial cells via qRT-PCR (*n* = 3), which were treated with Ibrutinib, Acalabrutinib or Zanubrutinib (2 μM). Data are shown as mean ± SEM; ** *p* < 0.01; *** *p* < 0.001; **** *p* < 0.0001, as calculated using one-way ANOVA with Tukey’s multiple comparisons test.

To reveal the molecular mechanisms leading to different activities against endothelial cells of BTKis, RNA-seq analyses were performed on HUVECs with or without treatment by Ibrutinib, Acalabrutinib or Zanubrutinib. An overall hierarchical clustering analysis was performed for all of the 1215 DEGs detected in the three comparisons, showing that the expression patterns of most DEGs under treatment with Ibrutinib or Zanubrutinib were significantly different to control group, whereas the expression pattern of DEGs in Acalabrutinib treatment group was similar to that of control group (Supplementary Figure S2A). Also, Ibrutinib and Zanubrutinib regulated 814 and 630 DEGs respectively. According to the KEGG classification, 42, 4, and 49 DEGs involved in cell growth and death were regulated by Ibrutinib, Acalabrutinib and Zanubrutinib, respectively (Supplementary Figure S3), from which 20, 2 and 14 apoptosis-related DEGs were obtained in each BTKi treatment group (Figure 2A), suggesting that Ibrutinib and Zanubrutinib regulated more apoptotic genes in HUVECs compared to Acalabrutinib, which may result in their higher cytotoxic activity on HUVECs. We next performed a hierarchical clustering analysis for the unions of apoptosis-related DEGs regulated by each BTKi (Figure 2B). Then, pro-apoptotic genes, *FOS* and *GADD45B*, and anti-apoptotic gene, *BCL2A1*, were screened and validated by qRT-PCR (Figure 2C), which indicated that Ibrutinib and Zanubrutinib dramatically modulated the expression of such genes, however, Acalabrutinib downregulated *BCL2A1* weakly and was not able to alter the expression of *GADD45B* and *FOS* significantly. Also, more effectiveness of Ibrutinib to upregulate *FOS* and *GADD45B* than Zanubrutinib may result in its higher toxicity on HUVECs than Zanubrutinib. These data suggested that Ibrutinib, Acalabrutinib and Zanubrutinib had different impacts on HUVECs, due to their distinct abilities to regulate apoptotic target genes.

### 3.3. BTKis Have Distinct Capacities to Induce Vascular Endothelial Dysfunction

**Figure 3:**
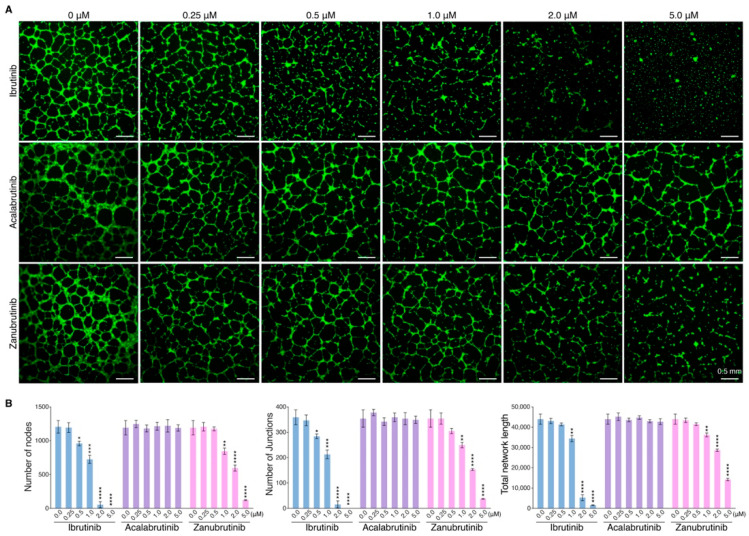
Effect of BTKis on endothelial network formation. (**A**) Endothelial cells were incubated for 8 h in Matrigel and treated with Ibrutinib, Acalabrutinib or Zanubrutinib in a dose-dependent manner and endothelial networks were observed under fluorescence microscopy after staining endothelial cells with Calcein AM. Scale bars, 0.5 mm. (**B**) Quantification of nodes, junctions and total network length of endothelial networks by ImageJ software (*n* = 3). Data are shown as mean ± SEM; * *p* < 0.05; ** *p* < 0.01; *** *p* < 0.001; **** *p* < 0.0001, as calculated using the Student’s *t*-test.

In addition to reveal the pro-apoptotic effects of BTKis on endothelial cells, we performed an in vitro angiogenesis assay based on Matrigel matrix to determine whether the BTKis could induce vascular endothelial dysfunction leading to vascular integrity disruption. HUVECs were pretreated with each BTKi in a dose-dependent manner for 8 h, and then were seeded on Matrigel matrix. The endothelial cell tube formation was observed 8 h later under fluorescence microscope after the alive cells were stained with Calcein AM (Figure 3A). ImageJ software was used to calculate the number of nodes, junctions and total network length of the vessels (Figure 3B). The results indicated that Ibrutinib had a strong capacity to disrupt vascular integrity even in low concentration, and vessels collapsed while HUVECs were treated with Zanubrutinib at high concentration, however, Acalabrutinib could not disrupt vascular integrity even at high concentration. These results indicated that Ibrutinib could disrupt vascular integrity efficiently, which might be potentially associated with its more risk of bleeding.

### 3.4. Ibrutinib Induces Vascular Endothelial Dysfunction via Upregulating BMP4

**Figure 4:**
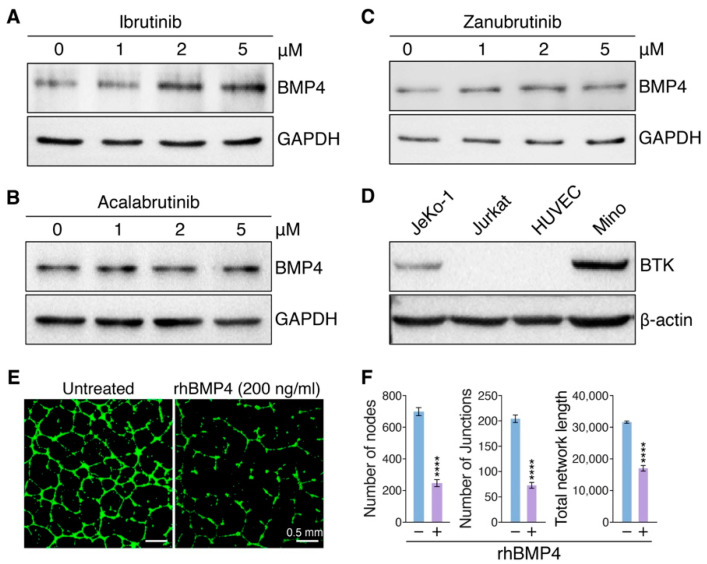
Ibrutinib-induced BMP4 disrupts vascular dysfunction. Analysis of protein expression of BMP4 in HUVECs. Endothelial cells were treated with Ibrutinib (**A**), Acalabrutinib (**B**) or Zanubrutinib (**C**) in a dose-dependent manner as indicated and immunoblot analysis of BMP4 expression was conducted. GAPDH was used as loading control. (**D**) Immunoblot analysis of BTK expression in HUVECs. Two MCL cell lines, JeKo-1 and Mino, were considered as positive controls of BTK expression, and a T-cell lymphoma cell line, Jurkat, was considered as negative control of BTK expression. β-actin was used as loading control. (**E**) The net-like structure of endothelial cells on Matrigel was decreased by the treatment of rhBMP4 (200 ng/mL), which was observed under fluorescence microscopy after staining endothelial cells with Calcein AM. Scale bars, 0.5 mm. (**F**) Quantification of nodes, junctions and total network length of endothelial networks from (**E**) by ImageJ software (*n* = 5). Data are shown as mean ± SEM; **** *p* < 0.0001, as calculated using the Student’s *t*-test.

Previous findings suggested that BMP4 could upregulate THBS1 expression and BMP4/THBS1 loop suppress tumor angiogenesis [23]. Based on our RNA-seq analysis, we found that BMP4 and THBS1 were upregulated more significantly in HUVECs treated with Ibrutinib than Zanubrutinib, however, Acalabrutinib had no such effect (Supplementary Figure S4A). The capacities of BTKis to modulate BMP4 and THBS1 expression were validated by qRT-PCR (Supplementary Figure S4B,C). We then aimed to reveal the distinct stimulation of BMP4 by each BTKi might differentially induce endothelial cell dysfunction. We firstly confirmed BTKi-mediated modulation of BMP4 expression by immunoblot analysis at protein levels (Figure 4A–C and Supplementary Figure S4D), which is consistent with RNA-seq and qRT-PCR data. Of note, immunoblot results showed that there is no BTK expression in HUVECs, indicating that BTKi-regulated BMP4 expression was in the BTK-independent pathway (Figure 4D). Next, angiogenesis assays showed that rhBMP4 protein treatment could disrupt vascular integrity at a concentration of 200 ng/mL (Figure 4E,F). These data indicated that Ibrutinib might induce endothelial dysfunction via upregulating BMP4.

### 3.5. Targeting BMP4 Inhibits the Capability of Ibrutinib to Vascular Endothelial Dysfunction

**Figure 5:**
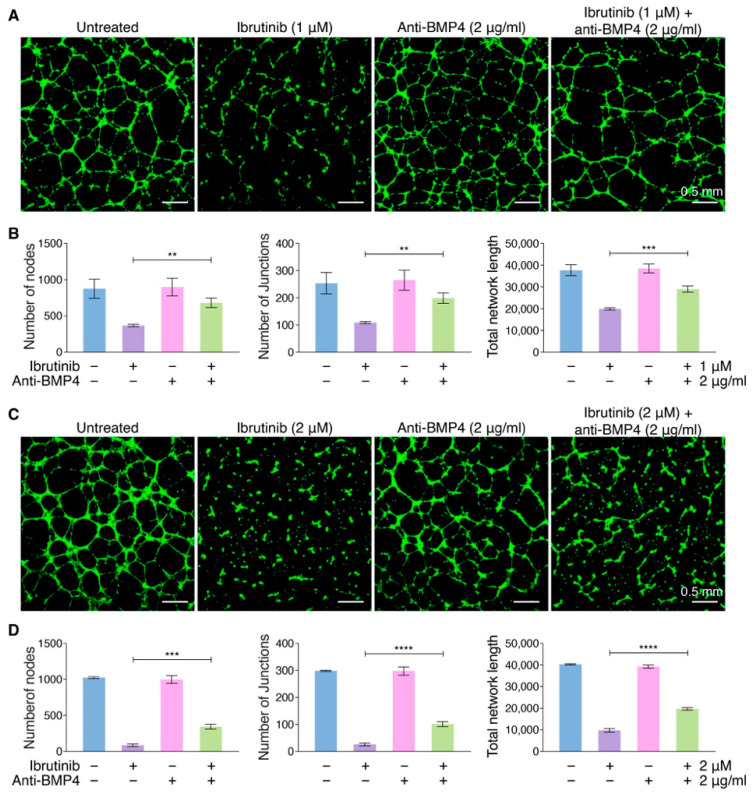
Anti-BMP4 mAb inhibits the capability of Ibrutinib to disrupt vascular dysfunction. (**A**) The net-like structure of endothelial cells on Matrigel under treatment with Ibrutinib (1 μM) and/or anti-BMP4 mAb (2 μg/mL), which was observed under fluorescence microscopy after staining with Calcein AM. Untreated endothelial cells were used as control. Scale bars, 0.5 mm. (**B**) The nodes, junctions and total network length of endothelial networks from (**A**) were quantified using the ImageJ software (*n* = 5). Data are shown as mean ± SEM; ** *p* < 0.01; *** *p* < 0.001, as calculated using one-way ANOVA with Tukey’s multiple comparisons test. (**C**) The net-like structure of endothelial cells on Matrigel under treatment with Ibrutinib (2 μM) and/or anti-BMP4 mAb (2 μg/mL), which was observed under fluorescence microscopy after staining endothelial cells with Calcein AM. Untreated endothelial cells were used as control. Scale bars, 0.5 mm. (**D**) The nodes, junctions and total network length of endothelial networks from (**C**) was quantified using the ImageJ software (*n* = 5). Data are shown as mean ± SEM; *** *p* < 0.001; **** *p* < 0.0001, as calculated using one-way ANOVA with Tukey’s multiple comparisons test.

Considering the more angiogenesis effect of Ibrutinib, we hypothesized that neutralization of BMP4 could be a potential strategy for reducing the strong suppressed angiogenesis of Ibrutinib on vascular integrity. Thus, we tested if anti-BMP4 mAb could impair the capacity of Ibrutinib to disrupt vascular integrity via neutralizing the function of BMP4 induced by Ibrutinib treatment. HUVECs were treated with or without Ibrutinib (1 μM or 2 μM) in the presence or absence of anti-BMP4 mAb (2 μg/mL), and anti-BMP4 mAb significantly counteracted the effect of Ibrutinib on vascular integrity disruption (Figure 5A–D). These data suggested that blocking BMP4 might be a potential strategy to relieve the bleeding effect caused by Ibrutinib-induced vascular damage.

### 3.6. Ibrutinib Inhibits Ovarian and Breast Cancer Progression In Vivo

**Figure 6:**
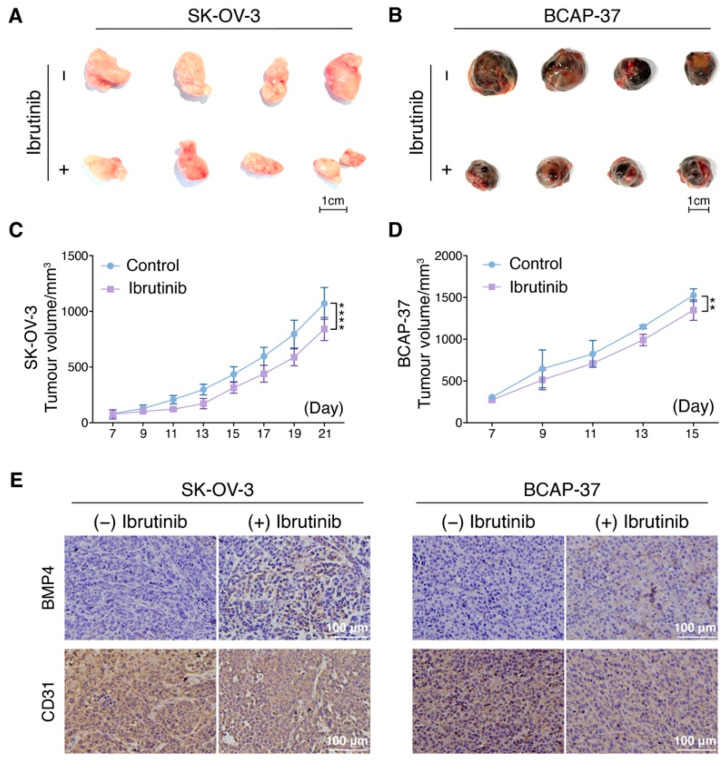
Solid tumor cell lines engrafted in nude mice are sensitive to Ibrutinib. (**A**) Representative SK-OV-3 tumor tissues of nude mice were shown, which were collected 21 days after subcutaneous injection of 1 × 10^7^ cancer cells. (**B**) Representative BCAP-37 tumor tissues of nude mice were shown, which were collected 15 days after subcutaneous injection of 1 × 10^7^ cancer cells. (**C**) Quantification of the SK-OV-3 tumor volume in animals treated with ibrutinib or vehicle at days 7, 9, 11, 13,15, 17, 19 and 21. (**D**) Quantification of the BCAP-37 tumor volume in animals treated with ibrutinib or vehicle at days 7, 9, 11, 13 and 15. (**C**,**D**) Tumor growth is represented as the mean ± SEM of the volume for the indicated number of animals; Significant changes between untreated or ibrutinib mice at the different points are labeled with ** *p* < 0.01; **** *p* < 0.0001; as calculated using two-way ANOVA with Tukey’s multiple comparisons test. (**E**) Representative images of immunohistochemistry analyses of tumors harvested from xenografted animals and stained for BMP4 and CD31. Scale bars, 100 μm.

To determine whether Ibrutinib may inhibit tumorigenesis in vivo, we evaluated the progression of ovarian cancer cells, SK-OV-3, and breast cancer cells, BCAP-37, in xenograft mouse models under Ibrutinib treatment versus no treatment. We found that the tumor volume, weight and growth curve were all significantly inhibited in the Ibrutinib treatment group compared to the NC group (Figure 6A–D). We then analyzed the expression of BMP4 and CD31 in these tumor tissues by immunohistochemistry analysis and found Ibrutinib treatment enhanced BMP4 expression and suppressed CD31 levels. These data suggested that Ibrutinib might inhibit tumorigenesis via impairing angiogenesis (Figure 6E).

## 4. Discussion

BTK is a major kinase in the BCR signaling pathway, which is highlighted by the clinical effectiveness of irreversible small-molecule BTKis, Ibrutinib, Acalabrutinib and Zanubrutinib, in B-cell malignancies. However, BTKis have an acceptable toxicity profile, and, among their effects, bleeding was observed in certain patients, especially Ibrutinib. Previous studies provided evidence that higher bleeding rates associated with Ibrutinib treatment were caused by the inhibition of TEC and SFK, contributing to platelet dysfunction [12,24], however, we cannot exclude that vascular stability disruption caused by destruction of the vascular endothelial cell junction may also result in bleeding. Thus, we examined the effects of BTKis on endothelial cells represented by HUVECs, and surprisingly found that Ibrutinib showed stronger suppressed angiogenesis to endothelial cells, followed by Zanubrutinib, and Acalabrutinib had slight lethality against endothelial cells, which conforms to their clinical manifestations in bleeding. These findings urged us to reveal the mechanisms driven by these three BTKis by RNA-seq analysis. Then, two classic pro-apoptotic genes, *FOS* and *GADD45B*, and classic anti-apoptotic gene *BCL2A1*, were identified, and the change of their expression levels might be critical for the apoptosis of endothelial cells induced by BTKis to distinct degrees. Ibrutinib and Zanubrutinib dramatically modulated the expression of *FOS*, *GADD45B* and *BCL2A1*, however, the expression levels of *BCL2A1* were reduced slightly by Acalabrutinib treatment and the expression of *GADD45B* and *FOS* were not controlled by Acalabrutinib, which might result in the higher toxicity of Ibrutinib and Zanubrutinib than Acalabrutinib. Also, higher toxicity of Ibrutinib against endothelial cells than that of Zanubrutinib might be caused by its better ability to upregulate the expression of *FOS* and *GADD45B*.

Except inducing apoptosis of endothelial cells directly, we hypothesized that BTKis may impair the angiogenesis ability of endothelial cells. Consequently, we performed in vitro angiogenesis assays to explore the influence of the three drugs on angiogenesis and found that Ibrutinib treatment was more effective to disrupt angiogenesis ability of endothelial cells in a dose-dependent manner, and vessels collapsed while endothelial cells were treated with Zanubrutinib at high concentration. However, Acalabrutinib had no effect on angiogenesis, even at high concentration. We also found the expression of angiogenesis marker CD31 was inhibited in the Ibrutinib treated group compared to the NC group in vivo. Such vascular damage effect of Ibrutinib was consistent with its more serious adverse event, bleeding, than those of Zanubrutinib and Acalabrutinib in patients under their treatment. Moreover, aggressive B-cell lymphoma phenotype is always accompanied with increased angiogenesis, and blockage of angiogenesis could dramatically inhibit lymphoma-cell growth in vivo [25], thus our finding also suggested that inhibition of microvascular formation in lymph nodes or bone marrow might be a novel mechanism for Ibrutinib in lymphoma treatments, and suggested an alternative role of Ibrutinib as a potential novel angiogenesis inhibitor for fighting cancers via blocking the growth of blood vessels supporting cancer-cell survival, which needs further investigation.

Our data showed that BMP4 and THBS1 were upregulated more significantly in endothelial cells treated with Ibrutinib than Zanubrutinib, however, Acalabrutinib had no such effect. We performed angiogenesis assays and confirmed that Ibrutinib-induced BMP4 protein impaired the vascular integrity, which is in line with previous findings that recombinant BMP4 protein treatment was able to decrease the net-like structure of HUVECs and HMVECs on Matrigel via upregulating THBS1, reported by Tsuchida R, et al. in 2014 [23]. Accordingly, we revealed that Ibrutinib inhibits tumor development, growth and angiogenesis in the solid tumor cell lines. Ibrutinib exerted these functions by upregulating the expression of BMP4. A previous study also supported our conclusion showing that BMP4 is required to remove the network of capillaries sprouts from the rat pupil to produce a clear cornea during embryonic development [26]. Moreover, we provided evidence that anti-BMP4 mAb could significantly counteract the negative effect of Ibrutinib on angiogenesis by neutralizing the function of BMP4, and because of the low BMP4 levels and high tube-formation capability of normal HUVECs, anti-BMP4 mAb did not affect tube formation of untreated HUVECs. Of note, Rothhammer T, et al. demonstrated a pro-angiogenic role of BMP4 by examining tube formation of human microvascular endothelial cells treated with BMP4 protein versus untreated cells in 2007 [27], and such controversial conclusion might be because microvascular endothelial cell line was used in their study, however, Tsuchida R, et al. and we used primary HUVECs cells with better tube-formation capability. In addition, Rezzola S, et al. addressed the pro-angiogenic activity of BMP4 by enhancing the migration of HUVECs at lower dose (50 ng/mL), however, higher dose of BMP4 (100 ng/mL) was not able to increase the migration. Also, the classic angiogenesis assay was not performed to evaluate tube-formation activity of HUVECs by Rezzola S, et al. [28].

In recent years, Ibrutinib has been proved as a therapeutic option against solid tumors. Ibrutinib could inhibit breast cancer cell-proliferation and metastasis by switching the phenotype of myeloid-derived suppressor cells to mature dendritic cells and enhancing the expression of major histocompatibility complex II in the orthotopic mouse model [29]. In pancreatic cancer, Ibrutinib inhibited tumor development by decreasing the phosphorylation of EGFR and reversed the upregulation of p-AKT and downstream genes induced by radiation [30]. Furthermore, Ibrutinib decreased BTK phosphorylation and Sox2/Bcl-xL expression in ovarian cancer, diminishing their self-renewal capacities and proportion of cancer stem cells [31]. Unfortunately, in phase III of the RESOLVE study (NCT02436668), Ibrutinib plus nab-Paclitaxel/Gemcitabine did not improve overall survival (median of 9.7 vs. 10.8 months; *p* = 0.3225) or progression-free survival (median 5.3 vs. 6.0 months; *p* < 0.0001) for patients with pancreatic ductal adenocarcinoma [32].A colorectal cancer (CRC) phase 1/2 study (NCT03332498) also discovered Ibrutinib 560 mg daily plus pembrolizumab 200 mg every 3 weeks appears to give limited anti-cancer activity in metastatic CRC, but the authors observed a significantly better safety profile than the phase III RESOLVE study [32], with 16 (42%) patients experiencing a grade 3/4 adverse effects [33]. In this study, we made a parallel analysis of Ibrutinib, Acalabrutinib and Zanubrutinib to uncover their potential similarities and differences in suppressing angiogenesis on human vascular endothelial cells. We found that Ibrutinib stronger suppressed angiogenesis on human vascular endothelial cells than Zanubrutinib and Acalabrutinib. Accordingly, we provided evidences that Ibrutinib suppressed the progression of ovarian and breast cancer cells in vivo by suppressing angiogenesis in a BTK-independent way due to its off-target effects.

## 5. Conclusions

Collectively, our findings illustrated the high capacity of Ibrutinib to induce apoptosis in primary human vascular endothelial cells in a BTK-independent manner, and revealed the distinct molecular mechanisms compared to Acalabrutinib and Zanubrutinib. Moreover, we identified Ibrutinib as a novel angiogenesis inhibitor via stimulating BMP4 expression leading to vascular endothelial dysfunction. Such findings suggested the potential application of Ibrutinib to treat angiogenesis-dependent cancers, such as ovarian cancer and breast cancer.

## Data Availability

The data presented in this study are available on request from the corresponding author.

## Supplementary Materials

The following supporting information can be downloaded at: https://www.mdpi.com/article/10.3390/pharmaceutics14091876/s1, Figure S1: BTKis differentially induced cell apoptosis in HUVECs; Figure S2: BTKi target genes identified by RNA-seq analysis in HUVECs; Figure S3: KEGG pathway analysis of BTKi-regulated target genes in HUVECs; Figure S4: RNA-seq, qRT-PCR and Immunoblot identified BTKis-induced BMP4 or THBS1 expression in HUVECs; Table S1: The primers used for qRT-PCR validation.
