# Supplementary material for: Ibrutinib Inhibits Angiogenesis and Tumorigenesis in a BTK-Independent Manner
Source: Pharmaceutics. 2022 Sep 5;14(9):1876. doi: 10.3390/pharmaceutics14091876 (PMC9506105; doi:10.3390/pharmaceutics14091876)
Supplement: Supplementary file 1 [file pharmaceutics-14-01876-s001.zip › pharmaceutics-1787011-supplementary.pdf]

Supplementary Material: Supplementary Tables and Figures

Ibrutinib Inhibits Angiogenesis and Tumorigenesis in a BTK-Independent Manner

Jia Liu [1,2,3,†], Zhuojun Liu [1,2,3,†], Jing Zhang [1,3], Xiaofang Chen [1,2], Junge Chen [1,3,], Linlin Sui [4,*] and Jian Yu [1,3,*]

[1] School of Engineering Medicine, Beihang University, Beijing 100083, China; dr_jialiu@buaa.edu.cn (J.L.); zhuojunl@buaa.edu.cn (Z.L.); jz2716@buaa.edu.cn (J.Z.); xfchen@buaa.edu.cn (X.C.); chenjg19@buaa.edu.cn (J.C.)
[2] School of Biological Science and Medical Engineering, Beihang University, Beijing 100083, China
[3] Beijing Advanced Innovation Center for Biomedical Engineering, Beihang University, Beijing 100083, China
[4] Core Lab Glycobiol & Glycoengn, College of Basic Medical Sciences, Dalian Medical University, Dalian 116000, China
* Correspondence: suill06@dmu.edu.cn (L.S.); yulab@buaa.edu.cn (J.Y.)
† These authors contributed equally to this work.

Supplementary Table

Table S1. The primers used for qRT-PCR validation.

Gene name		Primer sequence
GADD45B	Forward	5'-GCCAGGATCGCCTCACAGTGG
	Reverse	5'-GGATTTGCAGGGCGATGTCATC
FOS	Forward	5'-GCCTCTCTTACTACCACTCACC
	Reverse	5'-AGATGGCAGTGACCGTGGGAAT
BCL2A1	Forward	5'-GGATAAGGCAAAACGGAGGCTG
	Reverse	5'-CAGTATTGCTTCAGGAGAGATAGC
BMP4	Forward	5'-CTGGTCTTGAGTATCCTGAGCG
	Reverse	5'-TCACCTCGTTCTCAGGGATGCT
THBS1	Forward	5'-GCTGGAAATGTGGTGCTTGTCC
	Reverse	5'-CTCCATTGTGGTTGAAGCAGGC
GAPDH	Forward	5'-GTCTCCTCTGACTTCAACAGCG
	Reverse	5'-ACCACCCTGTTGCTGTAGCCAA

Supplementary Figures

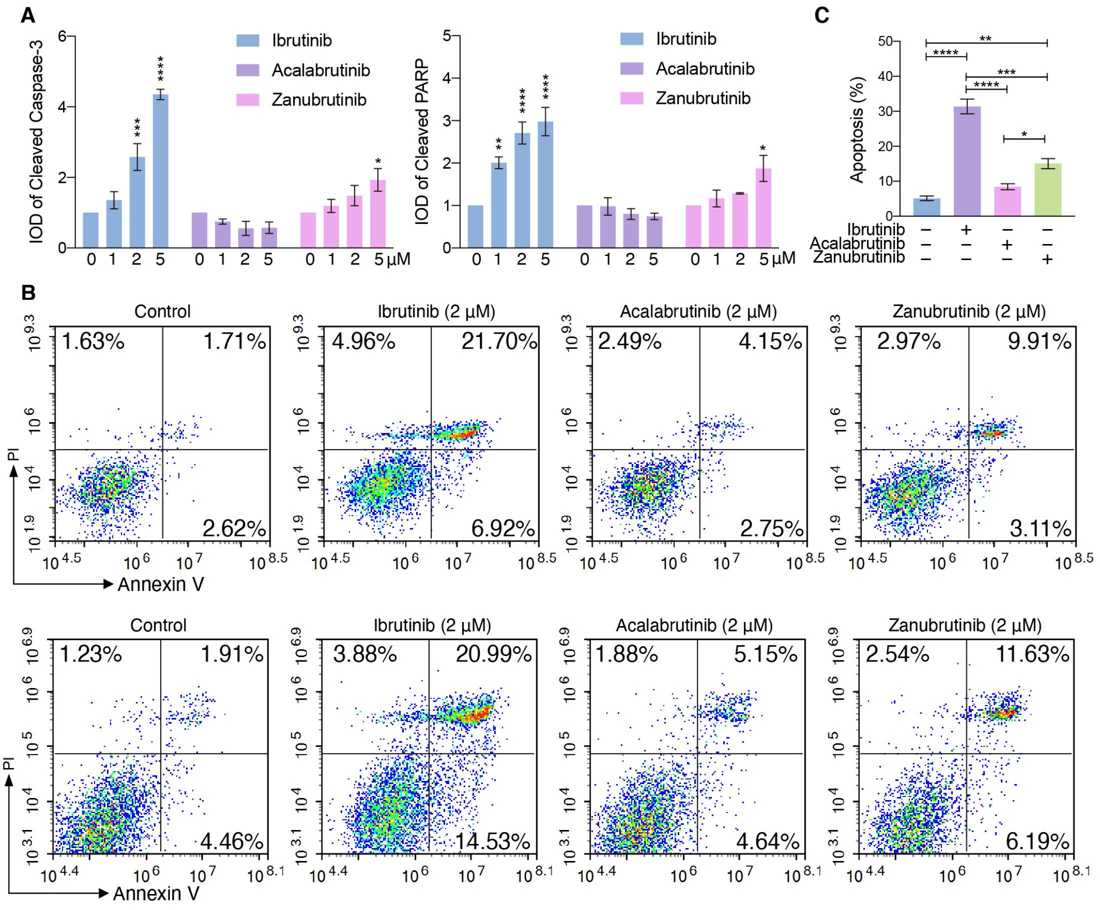

Figure S1. BTKis differentially induced cell apoptosis in HUVECs. **(A)** Each symbol represents the ratio of band IOD values for cleaved caspase-3 and cleaved PARP versus GAPDH normalized with respect to untreated samples (n = 3). Data are shown as mean ± SEM; *P < 0.05; **P < 0.01; ***P < 0.001; ****P < 0.0001, as calculated using one-way ANOVA with Tukey's multiple comparisons test. **(B)** Flow cytometry analysis of apoptosis induced by Ibrutinib, Acalabrutinib or Zanubrutinib at 48 h in HUVECs stained with Annexin V-FITC and PI, which were analyzed by NovoExpress Software. The percentages in the top right and bottom right of each pseudocolor plot indicate the proportion of Annexin V/PI-positive apoptotic cells. **(C)** Apoptotic percentage of (B) are depicted in the bar graph (n = 3). Data are shown as mean ± SEM; *P < 0.05; **P < 0.01; ***P < 0.001; ****P < 0.0001, as calculated using one-way ANOVA with Tukey's multiple comparisons test.

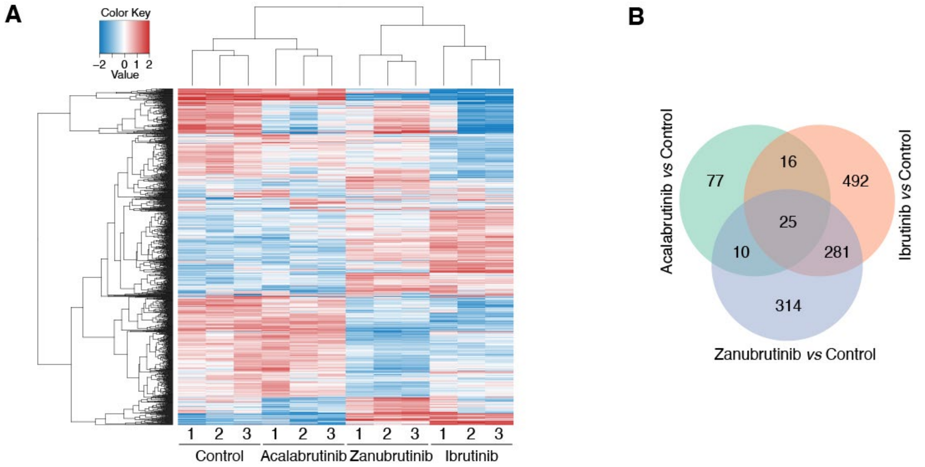

Figure S2. BTKi target genes identified by RNA-seq analysis in HUVECs. (A) Heatmap of DEGs regulated by Ibrutinib, Acalabrutinib and Zanubrutinib versus control in HUVECs. The color of each spot in the heatmap corresponds to log-transformed average relative expression levels of each gene in three samples by a gradient of color from blue (downregulation) to red (upregulation). **(B)** Venn diagram illustrating the number of genes commonly and differentially expressed in Ibrutinib, Acalabrutinib or Zanubrutinib compared to control in HUVECs.

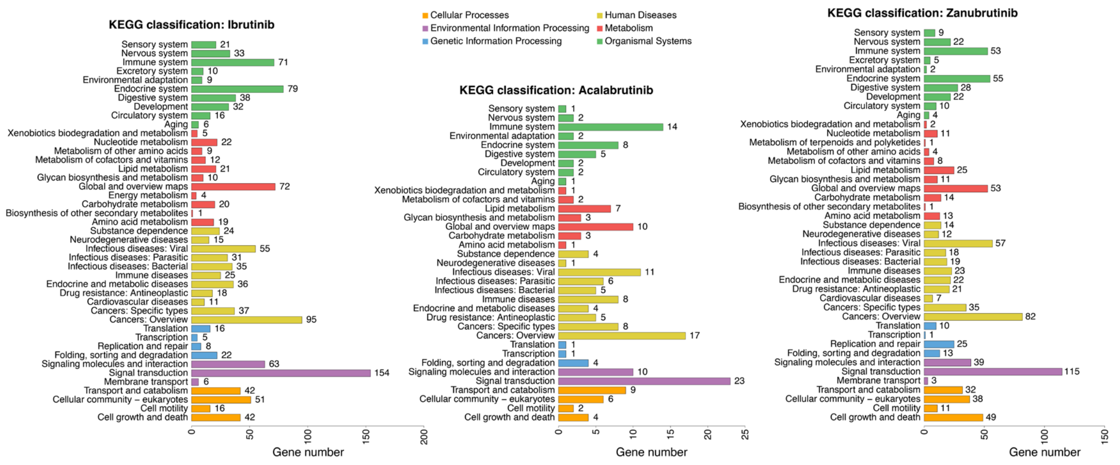

Figure S3. KEGG pathway analysis of BTKi-regulated target genes in HUVECs. Functional classification of KEGG pathway was analyzed by using the DEGs regulated by Ibrutinib, Acalabrutinib or Zanubrutinib in HUVECs. The bar length represents the observed number of genes within the respective KEGG pathway. The y-axis indicates the name of the KEGG pathways. The x-axis represents the observed number of genes annotated under that pathway.

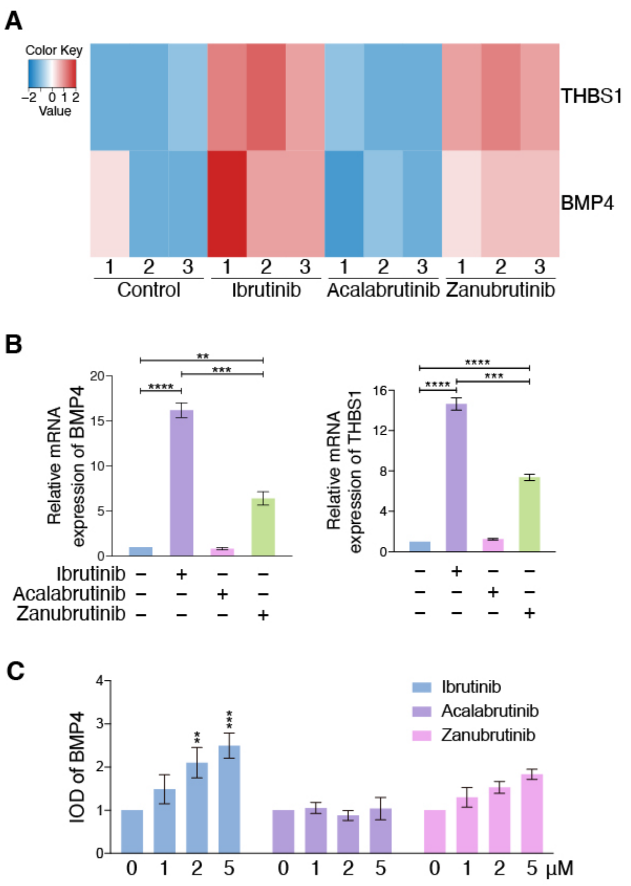

Figure S4. RNA-seq, qRT-PCR and Immunoblot identified BTKis-induced BMP4 or THBS1 expression in HUVECs. (A) Heatmap of *BMP4* and *THBS1* regulated by Ibrutinib, Acalabrutinib and Zanubrutinib versus control in HUVECs. Red represents upregulation and blue indicates downregulation. **(B)** Analysis of mRNA expression of *BMP4* and *THBS1* in endothelial cells via qRT-PCR (n = 3), which were treated with Ibrutinib, Acalabrutinib or Zanubrutinib (2 μM). Data are shown as mean ± SEM; **P < 0.01; ***P < 0.001; ****P < 0.0001, as calculated using one-way ANOVA with Tukey's multiple comparisons test. **(C)** Each symbol represents the ratio of band IOD values for BMP4 versus GAPDH normalized with respect to untreated samples (n = 3). Data are shown as mean ± SEM; **P < 0.01; ***P < 0.001, as calculated using one-way ANOVA with Tukey's multiple comparisons test.
